# *In Silico* Determination of the Effect of Multi-Target Drugs on Calcium Dynamics Signaling Network Underlying Sea Urchin Spermatozoa Motility

**DOI:** 10.1371/journal.pone.0104451

**Published:** 2014-08-27

**Authors:** Jesús Espinal-Enríquez, Alberto Darszon, Adán Guerrero, Gustavo Martínez-Mekler

**Affiliations:** 1 Instituto de Ciencias Físicas, Universidad Nacional Autónoma de México, Cuernavaca, Morelos, México; 2 Centro de Ciencias de la Complejidad, Ciudad Universitaria, México, México; 3 Instituto Nacional de Medicina Genómica, Arenal Tepepan, Tlalpan, México; 4 Departamento de Genética del Desarrollo y Fisiología Molecular, Instituto de Biotecnología, Universidad Nacional Autónoma de México, Cuernavaca, Morelos, México; 5 Laboratorio Nacional de Microscopía Avanzada, Instituto de Biotecnología, Universidad Nacional Autónoma de México, Cuernavaca, Morelos, México; 6 Centro Internacional de Ciencias, Cuernavaca, Morelos, México; Vanderbilt University Medical Center, United States of America

## Abstract

The motility of spermatozoa of both *Lytechinus pictus* and *Strongylocentrotus purpuratus* sea urchin species is modulated by the egg-derived decapeptide speract via an oscillatory [Ca^2+^]-dependent signaling pathway. Comprehension of this pathway is hence directly related to the understanding of regulated sperm swimming. Niflumic acid (NFA), a nonsteroidal anti-inflammatory drug alters several ion channels. Though unspecific, NFA profoundly affects how sea urchin sperm respond to speract, increasing the [Ca^2+^]*_i_* oscillation period, amplitude, peak and average level values of the responses in immobilized and swimming cells. A previous logical network model we developed for the [Ca^2+^] dynamics of speract signaling cascade in sea urchin sperm allows integrated dissection of individual and multiple actions of NFA. Among the channels affected by NFA are: hyperpolarization-activated and cyclic nucleotide gated Na^+^ channels (*HCN*), [Ca^2+^]-dependent Cl^−^ channels (*CaCC*) and [Ca^2+^]-dependent K^+^ channels (*CaKC*), all present in the sea urchin genome. Here, using our model we investigated the effect of blocking *in silico HCN* and *CaCC* channels suggested by experiments. Regarding *CaKC* channels, arguments can be provided for either their blockage or activation by NFA. Our study yielded two scenarios compliant with experimental observations: i) under *CaKC* inhibition, this [Ca^2+^]-dependent K^+^ channel should be different from the *Slo1* channel and ii) under activation of the *CaKC* channel, another [Ca^2+^] channel not considered previously in the network is required, such as the pH-dependent *CatSper* channel. Additionally, our findings predict cause-effect relations resulting from a selective inhibition of those channels. Knowledge of these relations may be of consequence for a variety of electrophysiological studies and have an impact on drug related investigations. Our study contributes to a better grasp of the network dynamics and suggests further experimental work.

## Introduction

**Figure 1:**
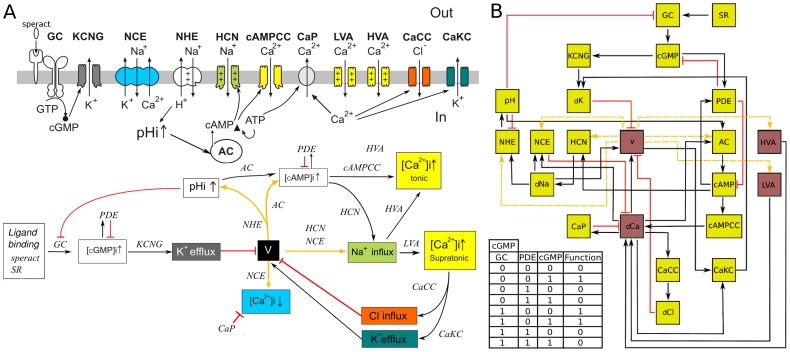
Speract-activated [Ca ^2+^] signaling pathway network model. A) Upper part: Schematic representation of the components of the signaling pathway triggered by speract in the sperm flagellum. Arrows traversing the membrane show ion fluxes. Arrows within the cell are indicative of causal relations. B) Bottom part: Signaling pathway operation diagram, black arrows correspond to activation, red lines to deactivation and yellow arrows can be activating or inhibitory depending on the relative state of the pathway elements being interconnected. Once speract binds to its receptor the several feedback loops are triggered according to the nature of the links involved. The concatenation of these loops leads to oscillatory stages of the whole pathway. The color code identifies corresponding upper and lower part components. Current models propose that the binding of speract to its receptor promotes the synthesis of cGMP that activates K^+^ selective and cyclic nucleotide-gated channels (KCNG) leading to membrane potential (V) hyperpolarization [3], [4], [7]–[11], [18]. This V change first induces an intracellular pH increase via a Na^+^/H^+^ exchanger (NHE) activation, [18], [51], [52], stimulates hyperpolarization-activated and cyclic nucleotide-gated channels (*HCN*) [53]–[55], removes the inactivation of voltage-gated 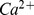 channels HVA and LVA [18], [56] (CaV), and facilitates 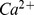 extrusion by Na^+^/Ca^2+^ exchangers (NCE) [51], [52]. The opening of *HCN* and the influx of Na^+^ contribute to V depolarization, and concomitant increases in 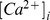 and 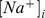 further depolarize V. It has been proposed that the 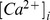 increases could lead to the opening of 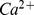-regulated Cl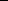 channels (*CaCC*) and/or 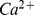-regulated K^+^ channels (*CaKC*), which would then contribute to hyperpolarize the V again, removing inactivation from CaV channels and opening *HCN* channels, [3], [4], [18]. It is thought that this series of events is then cyclically repeated generating a sequence of V-dependent turns. B) Network model of the signaling pathway. The network can be envisaged as a circuit where each node represents an element of the pathway and links, either in the form of arrows or lines, correspond to connections determined in the bottom part of (A). The activating or inhibitory nature of the yellow lines depends on the value of voltage (V). Yellow nodes represent binary nodes (0,1), and the four brown nodes are ternary nodes that can take values 0, 1 and 2. Changes in the node states are determined by the connected nodes by means of a regulatory function (or truth table). As an illustration we present the case of the cGMP shown at the bottom left of (B). The first 3 columns in this table contain all the possible activation states of the cGMP regulators: GC, which is an activator; PDE, an inhibitor and cGMP (cGMP is a self-regulator); the fourth column shows the values for the function that correspond to each combination of the regulators. Additional nomenclature note: Speract receptor (SR); guanylate cyclase (GC); unknown 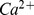 channels sensitive to cAMP (cAMPCC); 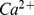 pump (CaP); dCa, dCl, dNa, dK are abbreviations for permeability changes in [Ca^2+^], [Cl^−^], [Na^+^] and [K^+^], respectively.

Fertilization is an important process in life. Reproductive success is attained by means of different strategies that increase the probability of gamete encounter. Several species, including sea urchins, produce spermatozoa with swimming patterns regulated by egg secretions. *Strongylocentrotus purpuratus* and *Lytechinus pictus* sea urchin spermatozoa swimming is modulated by speract, a decapeptide contained in the outer coating of the egg which diffuses in the sea [1], [2]. When these sperm detect speract by means of receptors along the flagellum, an intracellular signaling pathway that regulates fluctuations of the intracellular Ca^2+^ concentration ([Ca^2+^]*_i_*) is triggered. The pathway involves production of cyclic nucleotides and alterations in the ionic permeability of the plasma membrane (Fig 1A). These biochemical changes are timely translated to enhance the encounter of the sperm with the egg. Sea urchin spermatozoa swimming close to a surface describe circles, a very convenient trait to do imaging [3]–[5]. In the presence of speract gradients a cross-correlation of [Ca^2+^] oscillations and path curvatures takes place [3], [5]–[7]. Increases in the [Ca^2+^]*_i_* are associated with sharp turning events (high path curvature) that are interspersed with periods of straighter swimming episodes (low path curvature). This swimming pattern is common to a wide variety of organisms with external fertilization [3], [4], [8]–[16].

**Figure 2:**
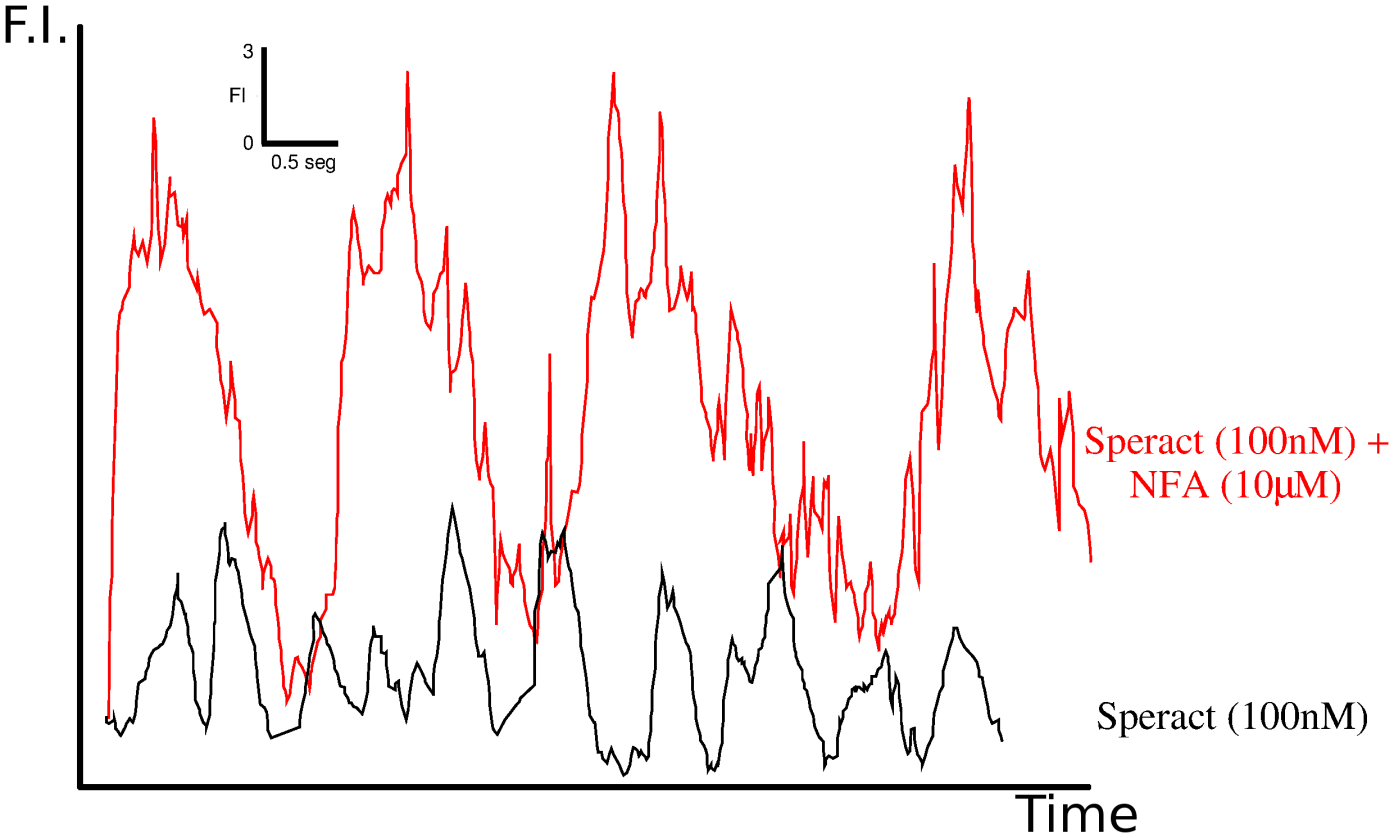
Niflumic acid increases the [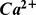]_*i*_ mean, maximum peak, amplitude and interval between successive [Ca^2+^] fluctuations. Experimental determination of the 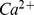-sensitive colorant Fluo-4 fluorescence in the flagellum, averaged along its length, of sperm exposed to 100 nM caged speract, in the absence of NFA (black trace) and the presence of 10 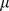
M of NFA (red trace). Concentration is measured in florescence intensity (F.I) units and time in seconds according to the scale shown in the inset on the upper right part of the figure. The figure is a modified version of the equivalent shown in [4].

Niflumic acid (NFA), a nonsteroidal anti-inflammatory drug, is able to block or modify several ion channels. Its lack of specificity, usually disadvantageous, turns out to be key to the profound effects it generates on how sea urchin sperm respond to speract. Immobilized *S. purpuratus* sperm exposed to NFA respond to speract with [Ca^2+^]*_i_* fluctuations that are larger, longer and with increased time intervals between them than control speract responses [17] (Fig 2). Furthermore, in *S. purpuratus* swimming sperm these alterations on flagellar [Ca^2+^]*_i_* dynamics caused by NFA increase the speractinduced flagellar asymmetry resulting in more pronounced and prolonged sharp turns [4]. In *L. pictus* swimming sperm NFA inhibits chemotaxis (also in *Arbacia Punctulata* [15]), without altering their ability to detect the chemoatractant gradient [8].

It is because of the findings mentioned above that it is very important and interesting to unravel how NFA achieves these responses. There are not many tools that efficiently allow analysis of multiple variables regulating a transduction path; certainly a network model of a signaling pathway is one. In this regard using this approach to examine how NFA may achieve its remarkable effects on the speract signaling cascade seems warranted. This Boolean network model has already shown its ability to uncover and predict properties of this signaling cascade. In a previous publication we presented a theory-experiment investigation where the model predicted the possible participation of a *CaKC* channel and a voltage dependent Ca^2+^ channel. Experiments simultaneously recording sperm [Ca^2+^]*_i_* and swimming trajectories, and using specific blockers for these channels gave results consistent with the model predictions [18].

Analysis of multi-target drugs provides a means for revealing interrelations and synergistic outcomes among various components of a biochemical pathway, evidencing systemic behaviors. The complexity of the experimental realization of such a research is evident. Taking into account our previous work with NFA [4], [8], [17] we focus on this inhibitor as an example. In particular, there is experimental evidence that NFA inhibits [Ca^2+^]-dependent [Cl^−^] Channels (*CaCC*) and Hyperpolarization-activated and Cyclic Nucleotide-Gated Channels (*HCN*), and stimulates [Ca^2+^]-dependent [K^+^] Channels (*CaKC*) of large conductance (BK_*Ca*_) [19]–[35]. However, it is not known if *CaKC* of small conductance (SK_*Ca*_) are blocked or activated by NFA [36]. In addition, blockage remains a possibility for other types of K^+^ channels such as K_*ATP*_ [37]. Hence, here we explore the consequences of assuming either activation or inhibition of *CaKC* channels.

Here we use our model to explore the effect of NFA on the speract signaling cascade of *S. purpuratus* and *L. pictus* sperm, always keeping in mind previous experimental results. We focus on four individual-cell quantities measured previously by Wood et al., in [4], namely, average [Ca^+^]_*i*_ level, amplitude, peak and frequency. We use them to compare the model predictions between oscillations in the wild type (WT) untreated network and in the NFA treated case, considering the two scenarios, depending on wheter NFA inhibits or activates *CaKC* channels.

## Methods and Models

### The logical network

The logical signaling network corresponding to the SASP, first introduced in [18], consists of nodes interlinked according to Fig 1A, representing the principal components involved in the signaling cascade: ion channel activities, intracellular ion and molecular concentrations and the membrane potential amongst others. The network is shown in Fig 1B and the nomenclature is explained in its figure caption. To analyze the dynamics of the network, we implemented a discrete formulation that is a generalization of the Boolean approach and that has proven to be revealing for the gene regulation dynamics of many systems [38]–[42], as well as other cell signaling networks [43]. In this approach, the dynamical state of the network consists of a set of 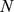 discrete variables 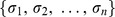, each representing the state of a node. For this particular network, most of the variables take on two values, 0 and 1, depending on whether the corresponding element is absent or present, closed or open, inactive or active, etc. However, an accurate description of the dynamical processes in the network required four nodes to be represented by three-state variables: the membrane potential (hyperpolarized 0, resting 1, and depolarized 2); the low and high threshold voltage-gated 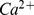 channels (inactive 0, closed 1, and open 2); and the intracellular [Ca^2+^] concentration 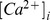 (basal 0, tonic 1 and supratonic 2). The state of each node 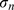 is determined by its set of regulators (which are some other nodes that also belong to the network). Let us denote as 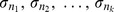 the 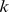 regulators of 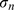. Then, at each time step the value of 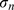 is given by
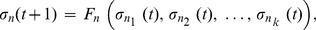
(1)where 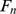 is a regulatory function constructed by taking into account the activating/inhibiting nature of the regulators. Each node has its own regulatory function. For the construction of these regulatory functions, we have made use of extensive biological knowledge, mainly of an electrophysiological nature, available to us in the literature and in our own laboratory. An illustration of such regulatory functions is given in Fig 1 for the cGMP node. For the case of [Ca^2+^], which is the main concern in our study, we have that it is regulated by 6 nodes (see origin of incoming arrows and red lines of *dCa* in Fig 1B). LVA, HVA and cAMP-dependent Ca^2+^ channels are activators, i. e., their activation at time *t*, will favor and increase in [Ca^2+^] at the subsequent time *t + 1*. There are also two inhibitory nodes: the Ca^2+^ pump and the Na^+^/Ca^2+^ exchanger which when activated at time *t* will favor a decrease in [Ca^2+^] at time *t + 1*. Finally the sixth node is the [Ca^2+^] node itself that acts as an inactivator, hence favoring a decrease in its value at the following time step. The balance among all the above inputs determined by physiological considerations is expressed in the form of a truth table with 432 rows, which constitutes its regulatory function. In the supplementary material S1 the regulatory functions for all the networks nodes are shown explicitly. With this model we can therefore observe *in silico* the effect of altering certain elements relevant to the pathway. In this paper we consider the case of NFA-sensitive channels: *HCN, CaKC* or *CaCC*. In order to test the effect of NFA on the network evolution, we proceeded with the deletion or activation of these channels according to the two scenarios mentioned above, individually, by pairs (*HCN* and *CaCC*, *HCN* and *CaKC*, *CaCC* and *CaKC*) and all three simultaneously. Within our model, node elimination takes place by assigning a zero value to all the regulatory entries in which it participates during the whole network evolution. Hence, each channel will be closed, even if its regulators are activated, thus simulating the blocking effect of NFA. Activation is put into practice by keeping the affected channel permanently open with a value of one. We calculated the modeled speract-triggered [Ca^2+^]_*i*_ fluctuations averaging the [Ca^2+^] node value over 10^5^ different initial conditions of the entire network (with all nodes present) and compared the result with equivalent calculations done on the treated networks. We repeatedly checked that after 10^5^ initial conditions the averaged [Ca^2+^] values essentially coincided with the complete initial condition calculations. With the averaged intracellular [Ca^2+^] ion concentration, determined from the model dynamics, which we shall denote by 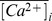, we have a representation with a finer resolution, more adequate for comparison with continuous experimental measurements.

### Steady state analysis

For this type of network (finite number of nodes which take a finite number of values), all initial conditions lead to a periodic behavior where the network configuration is replicated after a certain number of steps. The time required to reach this condition is known as the transient time; it is important to mention that, independently of the initial condition, it is shorter than 45 steps. The attractor is reached after 22 iterations and the number of iterations between the repeated configurations is the period. These periodic solutions are the attractors of the network dynamics. For the wild type (WT) network (with all nodes present), 88.9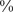 of all speract activated initial conditions lead to a period-4 attractor, while the remaining 11.1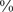 converge to a period-8 attractor [18]. The total amount of initial conditions which reach a particular attractor is called the basin of attraction. In order to understand the differences between the averaged [Ca^2+^] concentration *overline* 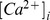 steady-state behavior of NFA-treated network and the WT, we calculate modifications of the network attractors after alteration of all combinations of NFA-sensitive channels, paying attention to the ensuing number of attractors and their associated periodicity. We make the comparisons between the characterization of the periodic behaviors of 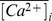 and the period of the network attractors. This is done by means of a Fourier spectrum analysis of 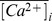 from which we can determine differences in the temporal behavior of WT and NFA-treated network dynamics. We should point out that we have explored the effect of small perturbations on the regulatory functions, such as modifications in the outcome of any row in the truth tables of all the nodes. This holds particularly well for [Ca^2+^] and is a measure of the high robustness of the dynamics we are implementing and hence of the reliability of our results.

## Results

### Individual Channel Results

**Figure 3:**
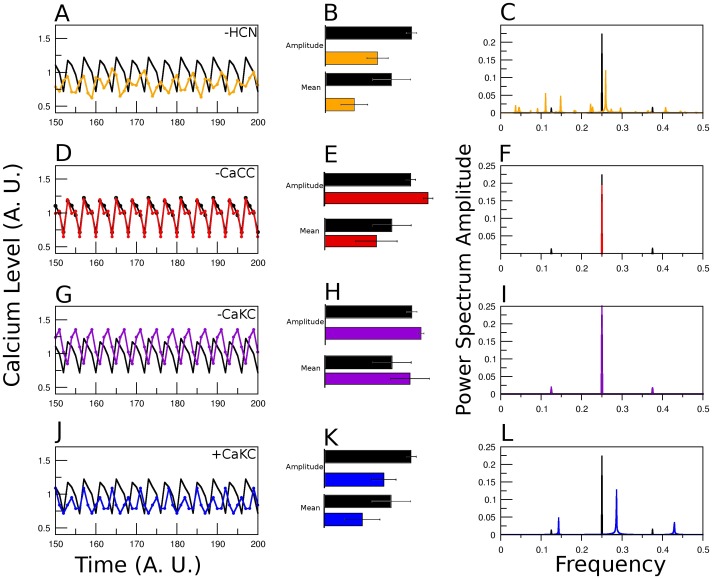
[Ca^2+^] dynamics comparison between WT network and blocking an individual NFA-sensitive channel. The [Ca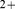] steady state fluctuations calculated form averaging over 100,000 initial conditions. For each initial condition, 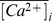 will take value 0 if it is in the basal state, 1 if it corresponds to a tonic state and 2 if it is supratonic. After averaging it will take values within the range [0–2] with a resolution of 10^5^. Concentration units are arbitrary, they comply with the above restrictions and are set for comparative purposes, time is measured by simulation steps and frequency refers to the fraction of a cycle covered by a simulation step, i.e cycles per simulation step. The values of 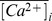 determined by the logical signaling network model, in the above mentioned A.U. (Arbitrary Units), are shown in black for the wild type case and in colors for the network with deletion of the node indicated in the figure: In the first column note A) for *HCN*, the loss of regularity; D) For *CaCC*, the increase in amplitude, and G) for *CaKC* on scenario 1, a higher average and peak values. J) *CaKC* on scenario 2 (activating *CaKC*), the decrease in peak, average and amplitude values. The central column (B, E, H and K) is the comparison between amplitude and mean of the 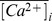 dynamics between the WT curve and those with altered channels. The colors are the same as in the first column. Right column (C, F, I and L): Fourier Power spectra obtained by the curves on the left.

In the first column of Fig 3, we show the 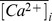 fluctuations after a transient of 150 time steps, determined by the model as a function of time, for the original network and for the network with the channel specified in the figure blocked (A, D and G) and activated (J). We should point out that an extensive statistical study shows that steady state conditions are attained with less than 150 iterations. Note the changes in the peak, mean and amplitude values, comparing the colored lines against the WT (black) curve. For a more quantitative comparison, in the second column (B, E, H and K), we show the bar plot of the average over 50 time steps of the amplitude and mean of the series shown to the left. In the third column (C, F, I and L) we focus on the temporal characterization of the series by means of the Fourier power spectrum of the 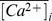 fluctuations. For each row, the channel altered is the same. Looking at the right column, the lines indicate the amplitude of the dominant frequencies in the Fourier decomposition of the 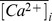 fluctuations depicted on the left column. The magnitude of each line indicates the weight of an underlying periodic contribution to the 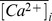 time series of a given Fourier decomposition mode. For the WT case, in Fig 3C, the first black line to the left, at the frequency 1/8 is the fundamental period-8 Fourier mode and reflects the contribution to the 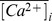 of the period-8 attractor obtained from the network dynamics. The third line at 3/8 is its harmonic. The middle line at frequency 1/4 is the result of the averaging procedure of the [Ca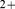] node network dynamics period-4 attractor together wtih the second harmonic of the period-8 attractor. We shall say that the 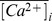 time series has a richer temporal behavior if its Fourier decomposition is more complex. Blockage of the *HCN* channel in our network model produces a more elaborate temporal behavior, which is registered in the Fourier power spectrum by the appearance of multiple lines that correspond to different modes in the Fourier decomposition. An opposite effect is observed with the blockage of the *CaCC* channel, where the Fourier decomposition consists of only one red line at the frequency value of 1/4 that corresponds to a unique period-4 attractor of the [Ca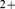] dynamics. For the case of *CaKC* blockage the Fourier spectrum remains unchanged with respect to the WT; this is consistent with the persistence in the blocked network of the WT attractors. Finally, activation of the *CaKC* channel produces 3 peaks at 1/7, 2/7 and 3/7 frequency values which are related to a period-7 attractor obtained by the network dynamics.

### Double Channel Results

**Figure 4:**
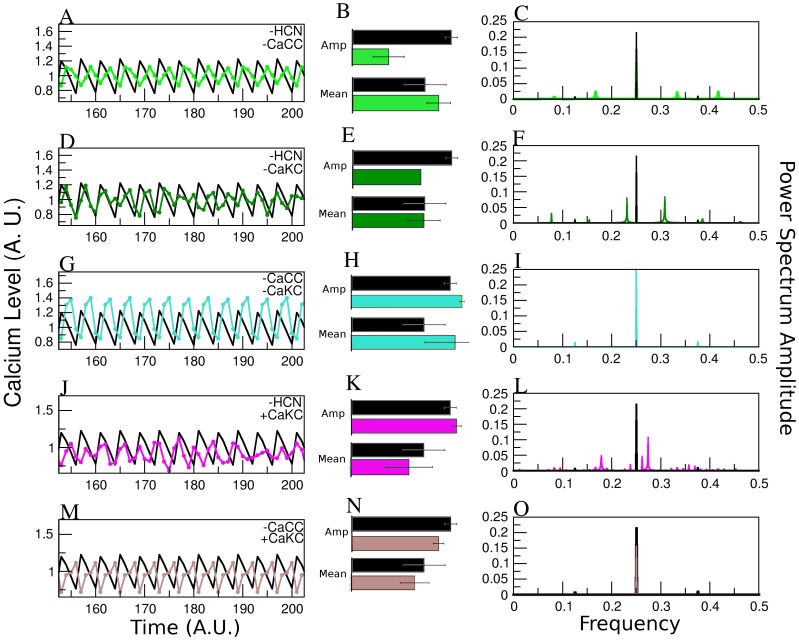
Effect on the Calcium dynamics of altering the different NFA-sensitive channels taken by pairs. Average over 100,000 initial conditions of the steady-state 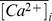 dynamics for the WT network is shown in black as in Figure 2. The columns are also distributed as in figure 2. A), B) and C) Blocking *HCN* and *CaCC* channels case (green line); D), E) and F) Blocking *HCN* and *CaKC* (dark green); G), H) and I) Blocking *CaCC*-*CaKC* (turquoise); J), K) and L) *HCN* blocked and *CaKC* activated; M), N) and O) *CaCC* blocked and *CaKC* activated. Notice that cases with *HCN* blocked produce non-regular [Ca^2+^] dynamics, opposite to the regularity generated by the *CaCC* blockage. Elimination of *CaKC* (scenario 1 in text) generates an elevation in [Ca^2+^] concentration compared with blockage of *CaCC* or *HCN*. Activation of *CaKC* (scenario 2) generates a decrease in 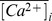 and peak, but the temporal behavior is more elaborate.

With regard to the temporal behavior, a similar analysis to the one performed for Fig 3, reaffirms the trends found from the individual channel alterations (Fig 4), namely: i) *HCN* blockage leads to a more complex Fourier Decomposition, consistent with a more elaborate behavior of the 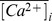 series shown on the left. ii) *CaCC* blockage reduces the Fourier components leading to a simpler periodic behavior. iii) *CaKC* activation enhances the effect of the concomitant deletion of either *HCN* or *CaCC*.

### Triple Channel Results

**Figure 5:**
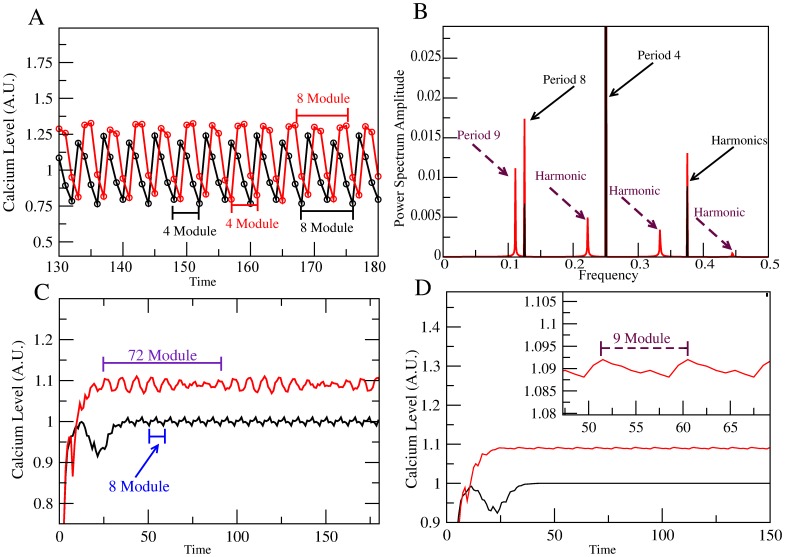
Effect of deletion of the three NFA-sensitive channels corresponding to scenario 1. A) Steady state time evolution of 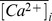 time series calculated as described in Fig 2. Average oscillations for the wild type are in black while oscillations under deletion of the *CaCC*-*CaKC*- *HCN* nodes are in red (the NFA-blocked network). For the wild type, two contiguous 4 element modules can be identified that together constitute a recurrent 8-step module. For the treated network different 4-step and 8-step modules are indicated in the figure. B) Fourier spectra calculated from 1000 steady-state steps of the 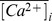 time series shown in (A). Period 4 and 8 Fourier modes and their harmonics are shown in black. For the NFA-blocked network the spectrum shown in red, determined from 1000 points of the steady-state [Ca^2+^] time series, is richer due to the appearance of a period 9 Fourier mode with its harmonics, besides the period 8 contribution. C) A four element running average of the series corresponding to (A) including the initial transient time manifests an underlying repeated 8 module for the wild type and 72 module for the NFA-blocked network case. This last module is the minimum common multiple (MCM) of the period 8 and 9 Fourier modes shown in (B). For the wild type case, a period-8 module surfaces which is the MCM of the period 4 and 8 Fourier modes shown in (B). D) Result of performing a running average as in C) using an 8 element window instead. Here the wild type oscillations are wiped out (black graph) while a 9 module envelop (in red) is evidenced for the triply blocked NFA treated case. The insert is an amplification that shows this behavior in detail.

**Figure 6:**
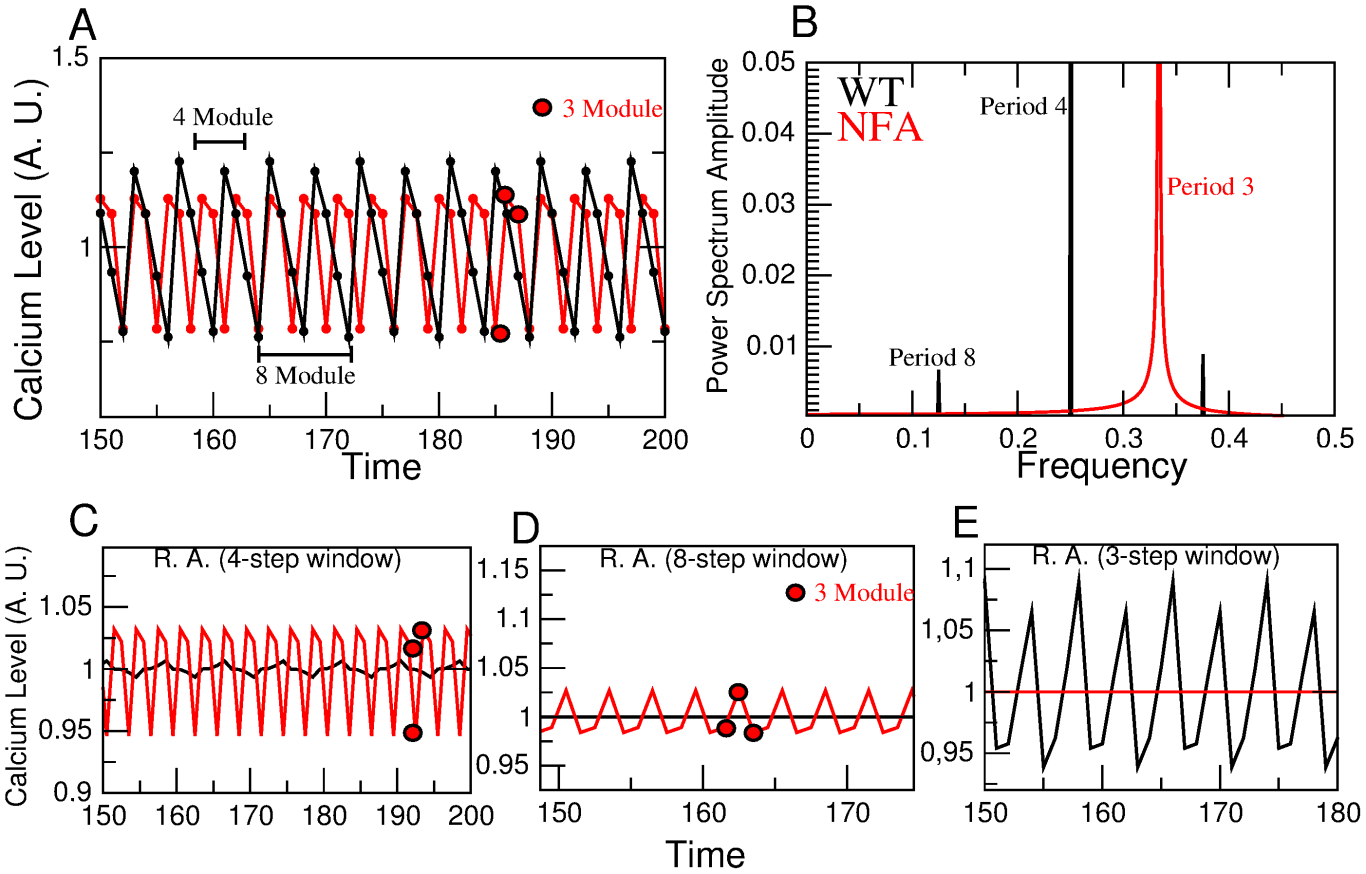
Effect of deletion of *HCN* and *CaCC* channels and activation of *CaKC*: scenario 2. A) Steady state time evolution of 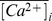 time series calculated as described in Fig 2. Average oscillations for the wild type are in black while oscillations under deletion of the *CaCC*- *HCN* as well as *CaKC* activation nodes are in red. Notice for the treated network, a 3-step module is indicated in the figure. B) Fourier spectra calculated from 1000 steady-state steps of the 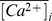 time series shown in A). Period 4 and 8 Fourier modes and their harmonics are shown in black. For the NFA-treated network the spectrum shown in red, determined from 1000 points of the steady-state [Ca^2+^] time series, presents a period-3 Fourier mode. C) A four element running average of the series corresponding to (A), manifests an underlying repeated 8 module for the wild type while the NFA-treated network preserves its period 3 oscillation. D) Result of performing a running average as in (C) using an 8 element window instead. Here the wild type oscillations are wiped out (black graph) while the 3 module envelop (in red) is evidenced for the triply blocked NFA treated case. E) Running average of size 3 produces the complete flattening of the NFA-treated network while period 8 persists in the WT.

With the joint alteration of the *HCN*-*CaCC*-*CaKC* channels the differences of the effect of blocking or activating the *CaKC* channel can be appreciated in Figs. 5 and 6 respectively. Fig 5A is a segment of the 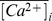 time series once steady-state conditions have been reached. Fig 5B is the Fourier spectrum calculated over 1000 steady-state time steps. Besides the coincidence of the WT period 4 and 8 peaks with their harmonics as before, a new feature that arises in the dynamics of the blocked system is the occurrence of a period-9 peak and one of its harmonics. This is an indication of a richer temporal behavior. If we perform a running average of the 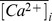 series over a window of 4 steps, the smaller fluctuations are dampened and the behavior of an envelope at larger scales becomes evident. This is shown in Fig 5C where a recurrent module of 72 time steps comes to light. Note that 72 is the minimum common multiple of the period 8 and 9 Fourier components, this is a manifestation of a period locking phenomena at the level of the 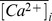 network attractors. If the running average is performed now over an 8 step window, then the WT 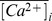 fluctuations are completely leveled out, as well as the period-8 component of the blocked case network, allowing for the period-9 component to surface (see Fig 5D). When activation of *CaKC* channel is considered, we encounter the behaviors shown in Fig 6 which are determined as in Fig 5. In Fig 6A the emergence of a 3 step module absent in the WT is noticeable. This new feature is reflected in the period 3 Fourier component shown in Fig 6B. When a 4 step average is performed, the WT is strongly smoothened and a period 8 structure emerges while the structure of NFA treated case remains similar (see Fig 6C). In Fig 6D we show that though the WT is completely flatten to its over-all average, under an 8 step running average, when the effect of NFA is taken into account a module 3 structure is preserved. For the converse case of averaging over 3 steps, it is the treated case that gets flatten while the 8 module consisting of two alternating 4 modules structure of the WT survives.

### Overall Picture

Given our *in silico* results, we can conclude from our data, the following: Blockage of the *HCN* channel affects mainly the temporal behavior of the [Ca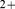] dynamics. This became evident in figures 3 and 4, where changes in the temporal structure of the Ca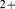 curve as well as the number of Fourier components can be observed. Another visible effect is the reduction of the 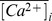 amplitude in all cases where *HCN* participates. When *CaCC* is blocked, the main effects are the increase of the amplitude in the 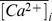 curve and a recovery of the regularity. Fourier components are reduced in all cases in which *CaCC* is altered. Regarding scenario 1, in which *CaKC* channel is inhibited, the principal effect is an elevation in the 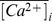 mean and maximum peak. In scenario 2, after activating the *CaKC* channel, the overall effect is quite similar to the one observed by *HCN* elimination: reduction in 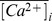 amplitude, peak and mean but, an increase in Fourier modes. Though all of these effects are reproduced under combinations of the above channel alterations, it is possible to establish the dominance of one channel alteration over another. For example, with regard to the fluctuation amplitude we have that the effect of deleting *HCN* overrides *CaCC* blockage, however, there is a recovery of regularity, situation in which the effect on temporal behavior of *CaCC* blockage overrides *HCN* elimination (Fig 4A). The same temporal observation is obtained by looking at the Fourier power spectrum for the case of the joint blockage of *HCN* and activation of *CaKC*. Both alterations result in an increase of the Fourier modes (Fig 4K) not only with regard to the WT but also to their action taken separately (Figs. 3C and 3L). This last result has a physiological explanation, because the effect of blockage of a channel which allows a cation entrance (*HCN*) or the activation of a channel which allows the cation extrusion (*CaKC*) will have a similar result in the regulation of membrane potential: shorter depolarizations.

Finally, the effect of altering the three channels together is analyzed. Here, we encounter the two scenarios mentioned previously, depending on whether *CaKC* channel is inhibited (Scenario 1, Fig 5) or activated (Scenario 2, Fig 6).

Overall our main findings are the following:

With respect to scenario 1, our analysis shows that only when NFA blocks simultaneously the three channels under consideration, we recover the experimental observations of [4] with regard to WT conditions for the model 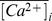 time steps, namely:

- The mean taken over the time increases (Fig 5A, C and D).
- The amplitude grows (Fig 5D).
- The peak values are higher (Figs 5A, C and D).
- The time interval between oscillations increases (Fig 5B).

In scenario 2 (Fig 6), the effect observed is a decrease in amplitude, period, and maximum peak, a trend opposite to the experiments. This behavior suggests the need of another [Ca^2+^] channel in the signaling pathway. An integrated presentation of our findings is condensed in Table 1. The red arrows correspond to those cases in which *CaKC* channel is activated (scenario 2). Table 1 clearly shows that within our modeling, experimental results from [4] can only be recovered under the joint NFA deletion of the three *HCN*, *CaCC* and *CaKC* channels. The first three columns of Table 1, state the cause-effect connections of individual channel deletions: the blockage of the *CaCC* channel increases 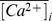 amplitude fluctuation, the blockage of the *CaKC* increases the 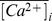 peak and mean values, and the blockage of the *HCN* channel confers a more elaborate temporal behavior.

**Table 1:** Effect of altering the NFA-sensitive channels in the Mathematical Model.

Channels	*HCN*	*CaCC*	*CaKC*	*HCN*-*CaCC*	*HCN*-*CaKC*	*CaCC*-*CaKC*	*All*
Mean	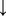	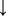	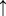 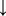	=	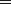 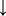	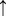 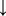	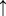 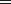
Amplitude	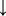	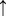	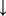 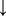	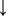	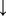 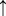	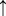 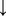	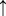 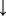
Peak	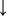	=	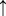 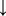	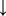	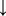 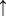	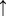 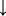	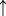 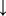
Fourier Mode Diversity	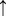	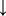	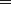 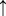	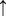	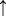 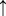	= 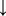	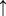 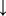

Compilation of results obtained by the logical network model altering one, two or the three NFA-sensitive channels in sea urchin spermatozoa. The non-bolded arrows correspond to scenario 1, in which the *CaKC* channel is inhibited. The bolded arrows indicate the alterations corresponding to scenario 2, with activation of *CaKC* channels.

## Discussion

### Summary

We developed a novel analysis of the effect of the multi-target drug niflumic acid, based on a logical network model of the speract-activated signaling pathway. We selectively modified the functionality of three ionic channels which are sensitive to NFA: *CaCC*, *HCN* and *CaKC*. We calculated [Ca^2+^] mean, amplitude, maximum peak and periodicity and determined the conditions under which accordance with previous experimental findings are attained. Other approaches have been done in terms of alterations of NFA in sea urchin spermatozoa [8], [44]. Although NFA is known to affect these three channels, we addressed the question of whether or not a concerted alteration takes place in the sea urchin, taking into account that most pharmacological results have been derived in mammalian channels [19], [21], [23], [24]. If *CaKC* channel is inhibited, we concluded that the experimental observations of [4] are retrieved only under a concomitant action of NFA on all three channels. The degree of synergy of this action remains to be elucidated. If *CaKC* channel is activated by NFA, the need of an additional [Ca^2+^] channel comes to light. Furthermore, along our investigation, indications of the relative importance of individual channel deletions surfaced. Our model predictions call for the implementation of experimental protocols for their corroboration. Knowledge of this type of cause-effect relations may be helpful for the understanding of the actions of diverse molecular compounds and might contribute to elucidate operating mechanisms of drugs.

### Physiological Context

**Figure 7:**
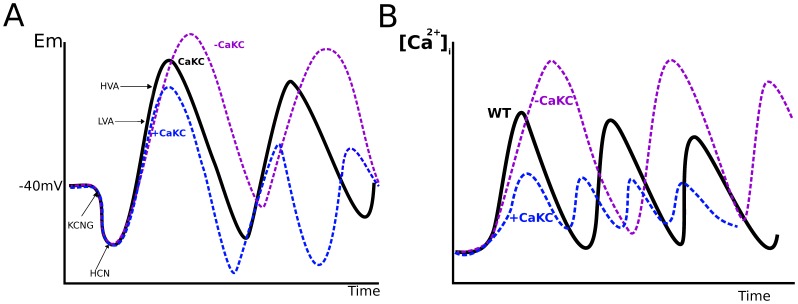
Schematic representation of the effect of deletion or activation of *CaKC* channels in the speract activated signaling pathway. A) the membrane potential dynamics. B), the [Ca^2+^] dynamics. For (A) the membrane potential is depicted in black for the WT network after speract activation. Hyperpolarization (lower than −40 mV) due to a 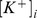 efflux via the KCNG channel, and the consequent opening of the voltage-dependent *HCN* channel which in turn depolarizes the sperm flagellum are shown. Depolarization (higher than −40 mV) opens LVA and HVA [Ca^2+^] channels. The increase in 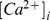 enhances depolarization. This last entrance of [Ca^2+^] opens the *CaCC* and *CaKC* channels with the corresponding influx of 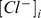 and efflux of 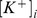 that hyperpolarize the membrane potential. All these steps are cyclically repeated causing the [Ca^2+^] oscillation pattern depicted in (B). If the effect of the drug is an inhibition of *CaKC* channels, this would cause a bigger depolarization, because a decrease in the 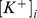 efflux. This is shown in the purple curve, notice that the amplitude is bigger than in the WT case. If the effect on *CaKC* is an activation instead, a bigger hyperpolarization is produced, due to the increase in the efflux of 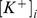. Depolarization takes less time and is smalles than in the WT case for the same reason (blue curve). For (B), the WT [Ca^2+^] dynamics is again depicted in black. Inhibition *CaKC* channels (purple curve), reduces the extrusion of 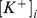 diminishing hyperpolarization hence delayning the closure of CaV channels. This will cause the elevation of 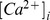 as well as the time between [Ca^2+^] peaks (the period). Overall, the activation of *CaKC* channels (blue curve), produces a shorter [Ca^2+^] oscillations due to the faster hyperpolarization, which in turn closes the CaV channels avoiding a big elevation of 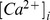.

The physiological effect that NFA may exert on the Ca^2+^-activated K^+^ channel *CaKC* in the speract-activated signaling pathway (SASP), either activating or inhibiting, can be explained in both cases (Fig 7):

- When *CaKC* is blocked, changes in membrane potential are attained, the depolarization is more pronounced owing to the reduction of 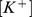 efflux (Fig 7A, violet line), hence, the voltage-dependent 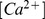 channels (CaVs) will remain open for a longer period; consequently, *CaKC* blockage (scenario 1) produces a bigger [Ca^2+^] curve (Fig 7B, violet line). Since there is pharmacological evidence of the presence of Slo1-type Ca^2+^-activated K^+^ channel in the SASP [18], and it is known that the Slo1 channel is activated by NFA [45], [46], the above line of reasoning could hold if we consider the participation of a different *CaCKC* such as SK_*Ca*_ or another K^+^ channel in the pathway. Given the results of the last column of table 1, these findings support this alternative, hinted by the experimental discrepancy mentioned in the introduction.
- In the case of scenario 2, after the first *KCNG*-dependent hyperpolarization, the activation of the *CaKC* will produce subsequent higher hyperpolarization values since this *CaKC* channel remains open. This in turn causes shorter lived depolarizations of a smaller magnitude, situation that leads to a premature closing of CaVs with the consequent decrease in 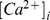. Under the assumption that NFA activates the *CaKC* channel, given that NFA is a non-specific drug, the inconsistency of the results between the 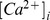 network dynamics with a *CaKC* channels activated *in silico* and experiments, favors the consideration of other [Ca^2+^] channels in the SASP sensitive to NFA, so far not present in our model. Suggestions along these lines are met by the sperm-specific *CatSper* channel, present in the sea urchin genome [47]–[49], which has recently been found to be a polymodal chemosensor in human sperm [50]. The pertinence and validity of this last suggestion, is a subject for further study.

Our investigation is closely related to the temporal behavior of the 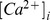 fluctuations. A proper understanding of temporal properties of the dynamics is fundamental for the comprehension of the link between internal biochemical oscillations that regulate molecular motors in the flagellum and the oscillatory space exploration of gradients of sperm activating peptides present in sea urchin swimming. Timing issues related to this interchange appear to be of importance for fertilization. In particular our study of the effect of NFA alterations on time-related characteristics may provide important issues on this link. Experimental analysis motivated by this work on the effect of NFA in the chemotactic response triggered by speract is provided in [8]. Finally, we would like to point out that the approach we have developed here can be used as a platform for the study of other biochemical signaling regulatory networks.

## Supporting Information

Material S1
**Regulatory tables for the Network Dynamics of the SASP.** Each one of the 21 text files which compose this compressed file, contains the name of the element (the file name), the name of its regulators, the whole combination of the dynamical states of those regulators (at time 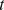) as well as the state of the regulated node (at time 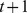), according to its logical rule (for more information, see Methods and Model section and figure 1).(ZIP)

## References

[1] Bentley J, Tubb D, Garbers D (1986) Receptor-mediated activation of spermatozoan guanylate cyclase. Journal of Biological Chemistry 261: 14859. 2876990

[2] Hansbrough JR, Garbers DL (1981) Speract. Purification and characterization of a peptide associated with eggs that activates spermatozoa. J Biol Chem 256: 1447–52. 6256397

[3] Wood CD, Darszon A, Whitaker M (2003) Speract induces calcium oscillations in the sperm tail. J Cell Biol 161: 89–101. 12695500 10.1083/jcb.200212053 PMC2172867

[4] Wood CD, Nishigaki T, Tatsu Y, Yumoto N, Baba SA, et al (2007) Altering the speract-induced ion permeability changes that generate flagellar Ca2+ spikes regulates their kinetics and sea urchin sperm motility. Dev Biol 306: 525–37. 17467684 10.1016/j.ydbio.2007.03.036

[5] Guerrero A, Nishigaki T, Carneiro J, Tatsu Y, Wood CD, et al (2010) Tuning sperm chemotaxis by calcium burst timing. Dev Biol 344: 52–65. 20435032 10.1016/j.ydbio.2010.04.013

[6] Guerrero A, Wood CD, Nishigaki T, Carneiro J, Darszon A (2010) Tuning sperm chemotaxis. Biochem Soc Trans 38: 1270–4. 20863297 10.1042/BST0381270

[7] Darszon A, Guerrero A, Galindo BE, Nishigaki T, Wood CD (2008) Sperm-activating peptides in the regulation of ion fluxes, signal transduction and motility. Int J Dev Biol 52: 595–606. 18649273 10.1387/ijdb.072550ad

[8] Guerrero A, Espinal J, Wood CD, Rendón JM, Carneiro J, et al.. (2013) Niflumic acid disrupts marine spermatozoan chemotaxis without impairing the spatiotemporal detection of chemoattractant gradients. J Cell Sci. 10.1242/jcs.121442 23418354

[9] Shiba K, Ohmuro J, Mogami Y, Nishigaki T, Wood CD, et al (2005) Sperm-activating peptide induces asymmetric flagellar bending in sea urchin sperm. Zoolog Sci 22: 293–299. 15795491 10.2108/zsj.22.293

[10] Kaupp UB, Solzin J, Hildebrand E, Brown JE, Helbig A, et al (2003) The signal flow and motor response controling chemotaxis of sea urchin sperm. Nat Cell Biol 5: 109–17. 12563276 10.1038/ncb915

[11] Kaupp UB, Kashikar ND, Weyand I (2008) Mechanisms of sperm chemotaxis. Annu Rev Physiol 70: 93–117. 17988206 10.1146/annurev.physiol.70.113006.100654

[12] Ward GE, Brokaw CJ, Garbers DL, Vacquier VD (1985) Chemotaxis of Arbacia punctulata spermatozoa to resact, a peptide from the egg jelly layer. J Cell Biol 101: 2324–2329. 3840805 10.1083/jcb.101.6.2324 PMC2113998

[13] Yoshida M, Murata M, Inaba K, Morisawa M (2002) A chemoattractant for ascidian spermatozoa is a sulfated steroid. Proc Natl Acad Sci U S A 99: 14831–14836. 12411583 10.1073/pnas.242470599 PMC137504

[14] Böhmer M, Van Q, Weyand I, Hagen V, Beyermann M, et al (2005) Ca2+ spikes in the flagellum control chemotactic behavior of sperm. EMBO J 24: 2741–52. 16001082 10.1038/sj.emboj.7600744 PMC1182239

[15] Alvarez L, Dai L, Friedrich BM, Kashikar ND, Gregor I, et al (2012) The rate of change in Ca(2+) concentration controls sperm chemotaxis. J Cell Biol 196: 653–663. 22371558 10.1083/jcb.201106096 PMC3307702

[16] Kashikar ND, Alvarez L, Seifert R, Gregor I, Jäckle O, et al (2012) Temporal sampling, resetting, and adaptation orchestrate gradient sensing in sperm. J Cell Biol 198: 1075–1091. 22986497 10.1083/jcb.201204024 PMC3444779

[17] Wood CD, Nishigaki T, Furuta T, Baba SA, Darszon A (2005) Real-time analysis of the role of Ca(2+) in flagellar movement and motility in single sea urchin sperm. J Cell Biol 169: 725–31. 15928204 10.1083/jcb.200411001 PMC2171626

[18] Espinal J, Aldana M, Guerrero A, Wood C, Darszon A, et al (2011) Discrete dynamics model for the speract-activated Ca2+ signaling network relevant to sperm motility. PLoS One 6: e22619. 21857937 10.1371/journal.pone.0022619 PMC3156703

[19] Pacaud P, Loirand G, Lavie JL, Mironneau C, Mironneau J (1989) Calcium-activated chloride current in rat vascular smooth muscle cells in short-term primary culture. Pflugers Arch 413: 629–636. 2726425 10.1007/BF00581813

[20] White MM, Aylwin M (1990) Niflumic and flufenamic acids are potent reversible blockers of Ca2(+)-activated Cl- channels in Xenopus oocytes. Mol Pharmacol 37: 720–724. 1692608

[21] Janssen LJ, Sims SM (1992) Acetylcholine activates non-selective cation and chloride conductances in canine and guinea-pig tracheal myocytes. J Physiol 453: 197–218. 1281502 10.1113/jphysiol.1992.sp019224 PMC1175553

[22] Akbarali HI, Giles WR (1993) Ca2+ and Ca(2+)-activated Cl- currents in rabbit oesophageal smooth muscle. J Physiol 460: 117–133. 7683715 10.1113/jphysiol.1993.sp019462 PMC1175204

[23] Espinosa F, de la Vega-Beltrán JL, López-González I, Delgado R, Labarca P, et al (1998) Mouse sperm patch-clamp recordings reveal single Cl- channels sensitive to niflumic acid, a blocker of the sperm acrosome reaction. FEBS Lett 426: 47–51. 9598976 10.1016/s0014-5793(98)00305-6

[24] Greenwood IA, Large WA (1995) Comparison of the effects of fenamates on Ca-activated chloride and potassium currents in rabbit portal vein smooth muscle cells. Br J Pharmacol 116: 2939–2948. 8680728 10.1111/j.1476-5381.1995.tb15948.x PMC1909225

[25] Hogg RC, Wang Q, Large WA (1994) Action of niflumic acid on evoked and spontaneous calcium-activated chloride and potassium currents in smooth muscle cells from rabbit portal vein. Br J Pharmacol 112: 977–984. 7921628 10.1111/j.1476-5381.1994.tb13177.x PMC1910202

[26] Satoh TO, Yamada M (2001) Niflumic acid reduces the hyperpolarization-activated current (I(h)) in rod photoreceptor cells. Neurosci Res 40: 375–381. 11463484 10.1016/s0168-0102(01)00252-8

[27] Cheng L, Sanguinetti MC (2009) Niflumic acid alters gating of HCN2 pacemaker channels by interaction with the outer region of S4 voltage sensing domains. Mol Pharmacol 75: 1210–1221. 19218366 10.1124/mol.108.054437 PMC2672808

[28] Gögelein H, Dahlem D, Englert HC, Lang HJ (1990) Flufenamic acid, mefenamic acid and niflumic acid inhibit single nonselective cation channels in the rat exocrine pancreas. FEBS Lett 268: 79–82. 1696554 10.1016/0014-5793(90)80977-q

[29] Farrugia G, Rae JL, Sarr MG, Szurszewski JH (1993) Potassium current in circular smooth muscle of human jejunum activated by fenamates. Am J Physiol 265: G873–G879. 8238516 10.1152/ajpgi.1993.265.5.G873

[30] Gribkoff VK, Lum-Ragan JT, Boissard CG, Post-Munson DJ, Meanwell NA, et al (1996) Effects of channel modulators on cloned large-conductance calcium-activated potassium channels. Mol Pharmacol 50: 206–217. 8700114

[31] Hu H, Tian J, Zhu Y, Wang C, Xiao R, et al (2010) Activation of TRPA1 channels by fenamate nonsteroidal anti-inflammatory drugs. Pflugers Arch 459: 579–592. 19888597 10.1007/s00424-009-0749-9 PMC2828537

[32] Wiemuth D, Gründer S (2011) The pharmacological profile of brain liver intestine Na+ channel: inhibition by diarylamidines and activation by fenamates. Mol Pharmacol 80: 911–919. 21828194 10.1124/mol.111.073726

[33] Busch AE, Herzer T, Wagner CA, Schmidt F, Raber G, et al (1994) Positive regulation by chloride channel blockers of IsK channels expressed in Xenopus oocytes. Mol Pharmacol 46: 750–753. 7969055

[34] Peretz A, Degani N, Nachman R, Uziyel Y, Gibor G, et al (2005) Meclofenamic acid and diclofenac, novel templates of KCNQ2/Q3 potassium channel openers, depress cortical neuron activity and exhibit anticonvulsant properties. Mol Pharmacol 67: 1053–1066. 15598972 10.1124/mol.104.007112

[35] Fernandez D, Sargent J, Sachse FB, Sanguinetti MC (2008) Structural basis for ether-a-go-go-related gene K+ channel subtype-dependent activation by niflumic acid. Mol Pharmacol 73: 1159–1167. 18218980 10.1124/mol.107.043505 PMC2493422

[36] Yang D, Arifhodzic L, Ganellin CR, Jenkinson DH (2013) Further studies on bis-charged tetraaza-cyclophanes as potent inhibitors of small conductance Ca(2+)-activated K+ channels. Eur J Med Chem 63: 907–923. 23685886 10.1016/j.ejmech.2013.02.029

[37] Grover GJ, D'Alonzo AJ, Sleph PG, Dzwonczyk S, Hess TA, et al (1994) The cardioprotective and electrophysiological effects of cromakalim are attenuated by meclofenamate through a cyclooxygenase-independent mechanism. J Pharmacol Exp Ther 269: 536–540. 8182522

[38] Kauffman SA (1969) Metabolic stability and epigenesis in randomly constructed genetic nets. J Theor Biol 22: 437–67. 5803332 10.1016/0022-5193(69)90015-0

[39] Espinosa-Soto C, Padilla-Longoria P, Alvarez-Buylla ER (2004) A gene regulatory network model for cell-fate determination during Arabidopsis thaliana flower development that is robust and recovers experimental gene expression profiles. Plant Cell 16: 2923–39. 15486106 10.1105/tpc.104.021725 PMC527189

[40] Albert R, Othmer HG (2003) The topology of the regulatory interactions predicts the expression pattern of the segment polarity genes in Drosophila melanogaster. J Theor Biol 223: 1–18. 12782112 10.1016/s0022-5193(03)00035-3 PMC6388622

[41] Huang S, Ingber DE (2000) Shape-dependent control of cell growth, differentiation, and apoptosis: switching between attractors in cell regulatory networks. Exp Cell Res 261: 91–103. 11082279 10.1006/excr.2000.5044

[42] Li F, Long T, Lu Y, Ouyang Q, Tang C (2004) The yeast cell-cycle network is robustly designed. Proc Natl Acad Sci U S A 101: 4781–6. 15037758 10.1073/pnas.0305937101 PMC387325

[43] Morris MK, Saez-Rodriguez J, Sorger PK, Lauffenburger DA (2010) Logic-based models for the analysis of cell signaling networks. Biochemistry 49: 3216–24. 20225868 10.1021/bi902202q PMC2853906

[44] Aguilera LU, Galindo BE, Sánchez D, Santillán M (2012) What is the core oscillator in the speract-activated pathway of the Strongylocentrotus purpuratus sperm flagellum? Biophys J 102: 2481–2488. 22713563 10.1016/j.bpj.2012.03.075 PMC3368143

[45] Li L, Ma KT, Zhao L, Si JQ (2008) Niflumic acid hyperpolarizes the smooth muscle cells by opening BK(Ca) channels through ryanodine-sensitive Ca(2+) release in spiral modiolar artery. Sheng Li Xue Bao 60: 743–750. 19082430

[46] Ottolia M, Toro L (1994) Potentiation of large conductance KCa channels by niflumic, flufenamic, and mefenamic acids. Biophys J 67: 2272–2279. 7535111 10.1016/S0006-3495(94)80712-X PMC1225611

[47] Liu J, Xia J, Cho KH, Clapham DE, Ren D (2007) CatSperbeta, a novel transmembrane protein in the CatSper channel complex. J Biol Chem 282: 18945–18952. 17478420 10.1074/jbc.M701083200

[48] Okamura Y, Nishino A, Murata Y, Nakajo K, Iwasaki H, et al (2005) Comprehensive analysis of the ascidian genome reveals novel insights into the molecular evolution of ion channel genes. Physiol Genomics 22: 269–282. 15914577 10.1152/physiolgenomics.00229.2004

[49] Darszon A, Nishigaki T, Beltran C, Treviño CL (2011) Calcium channels in the development, maturation, and function of spermatozoa. Physiol Rev 91: 1305–1355. 22013213 10.1152/physrev.00028.2010

[50] Brenker C, Goodwin N, Weyand I, Kashikar ND, Naruse M, et al (2012) The CatSper channel: a polymodal chemosensor in human sperm. EMBO J 31: 1654–1665. 22354039 10.1038/emboj.2012.30 PMC3321208

[51] Nishigaki T, Wood CD, Tatsu Y, Yumoto N, Furuta T, et al (2004) A sea urchin egg jelly peptide induces a cGMP-mediated decrease in sperm intracellular Ca(2+) before its increase. Dev Biol 272: 376–88. 15282155 10.1016/j.ydbio.2004.04.035

[52] Nishigaki T, Zamudio FZ, Possani LD, Darszon A (2001) Time-resolved sperm responses to an egg peptide measured by stopped-flow fluorometry. Biochem Biophys Res Commun 284: 531–535. 11394914 10.1006/bbrc.2001.5000

[53] Gauss R, Seifert R, Kaupp UB (1998) Molecular identification of a hyperpolarization-activated channel in sea urchin sperm. Nature 393: 583–7. 9634235 10.1038/31248

[54] Lee HC, Garbers DL (1986) Modulation of the voltage-sensitive Na+/H+ exchange in sea urchin spermatozoa through membrane potential changes induced by the egg peptide speract. J Biol Chem 261: 16026–32. 2430965

[55] Rodríguez E, Darszon A (2003) Intracellular sodium changes during the speract response and the acrosome reaction in sea urchin sperm. J Physiol 546: 89–100. 12509481 10.1113/jphysiol.2002.030510 PMC2342476

[56] Strünker T, Weyand I, Bönigk W, Van Q, Loogen A, et al (2006) A K+-selective cGMP-gated ion channel controls chemosensation of sperm. Nat Cell Biol 8: 1149–54. 16964244 10.1038/ncb1473

